# Supplementary material for: Lipid transfer from plants to arbuscular mycorrhiza fungi
Source: eLife. 2017 Jul 20;6:e29107. doi: 10.7554/eLife.29107 (PMC5559270; doi:10.7554/eLife.29107)
Supplement: Supplementary file 1. — DOI: http://dx.doi.org/10.7554/eLife.29107.045 [file elife-29107-supp1.docx]

**Table S1. Mutations in *DIS* and *DIS-LIKE* identified by TILLING or in a LORE1 insertion collection**.

| **Allele** | **Line ID** | **aa change / insertion** | **Source** |
| --- | --- | --- | --- |
|  |  |  |  |
| *dis-1* | SL0154-N | G190R | (Groth et al., 2013) |
| *dis-2* | 30035849 | LORE1 intron 2 insertion | (Małolepszy et al., 2016) |
| *dis-3* | SL4113-1 | T221I | RevGen, UK |
| *dis-4* | SL0614-1 | G314D | RevGen, UK |
| *dis-5* | SL5510-1 | E338K | RevGen, UK |
| *dis-6* | SL0494-1 | P376L | RevGen, UK |
|  |  |  |  |
| *disl-1* | SL3509-1 | P61S | RevGen, UK |
| *disl-2* | 30034395 | LORE1 exon 2 insertion | (Małolepszy et al., 2016) |
| *disl-3* | SL1481-1 | D109N | RevGen, UK |
| *disl-4* | SL5555-1 | G176E | RevGen, UK |
| *disl-5* | SL1474-1 | G180E | RevGen, UK |
| *disl-6* | SL4156-1 | V193M | RevGen, UK |

Groth M, Kosuta S, Gutjahr C, Haage K, Hardel SL, Schaub M, Brachmann A, Sato S, Tabata S, Findlay K, et al. 2013. Two *Lotus japonicus* symbiosis mutants impaired at distinct steps of arbuscule development. *The Plant Journal* **75**: 117-129. 10.1111/tpj.12220.

Małolepszy A, Mun T, Sandal N, Gupta V, Dubin M, Urbański D, Shah N, Bachmann A, Fukai E, Hirakawa H, et al. 2016. The LORE1 insertion mutant resource. *Plant Journal*: DOI: 10.1111/tpj.13243. 10.1111/tpj.13243.
