# Supplementary material for: Lipid transfer from plants to arbuscular mycorrhiza fungi
Source: eLife. 2017 Jul 20;6:e29107. doi: 10.7554/eLife.29107 (PMC5559270; doi:10.7554/eLife.29107)
Supplement: Supplementary file 2. — DOI: http://dx.doi.org/10.7554/eLife.29107.046 [file elife-29107-supp2.docx]

**Table S2. Primers used in this study.**

| **Purpose** | **Name** | **Sequence** |
| --- | --- | --- |
| g*DIS* cloning for p*DIS:gDIS* | SH71  SH72 | CACCGGAACGGGACAAAAGACTCC  TTAGGGCCTGAATGGAGCAAAGACAA |
| p*DIS* cloning for p*DIS:GUS* | SH94  SH104 | ATTTAAGCTTGGAACGGGACAAAAGACTCC  AATCAGGATCCTGTTCAATGTGTCTGTGGCA |
| *DIS* cloning for *DIS-RFP* localization in *N. benthamiana* | SH93  SH92 | CACCATGGCAAGCATTGCTGGTTC  GGGCCTGAATGGAGCAAAGACAAC |
| p*LjPT4* cloning for p*PT4:DIS* | CG466  CG467 | TTTGGTCTCTGCGGGGACTCAAGAAACCATGCTATC  TTTGGTCTCTCAGACTTGAACGATGTCGATTTAGTTTG |
| *DIS/dis-1* frag.1 cloning for p*PT4:DIS/dis-1* | SH124  SH125 | ATGAAGACTTTACGGGTCTCACACCATGGCAAGCATTGCTGGTTC  TTGAAGACTTTTCGATTTCAGGGCTCTCTTTGTTACCTGATGACAACAAGCACCCTTTTGG |
| *DIS/dis-1* frag.1 cloning for p*PT4:DIS/dis-1* | SH126  SH127 | TTGAAGACTTCGAAACCCTGATGATTATT  ATGAAGACTTCAGAGGTCTCACCTTGGGCCTGAATGGAGCAA |
| p*DIS* cloning for p*DIS:AtKASI* | SH122  SH123 | ATGAAGACTTTACGGGTCTCAGCGGGGAACGGGACAAAAGACTCC  ATGAAGACTTCAGAGGTCTCAGGTGTGTTCAATGTGTCTGTGG |
| p*AtKASI* cloning for p*AtKASI:LjDIS* | SH113  SH109 | TTGGTCTCACACCGAGTCACAAAGATGCTATCG  GGTCTCACCATGGTGGATCCAGAAATTGAGAG |
| *3’UTR AtKASI* cloning for p*AtKASI:LjDIS* | SH118  SH119 | TGAGGTCTCGTTTCTTCATACCTTTTAGATTC  TGAGGTCTCGCCTTCAGTATAAATCTAATTTCTTC |
| g*DIS* frag.1 cloning for p*AtKASI:LjDIS* | SH110  SH114 | TGAGGTCTCTATGGCAAGCATTGCTGGTTCATG  TGAGGTCTCTTTCGATTTCAGGGCTCTCT |
| g*DIS* frag.2 cloning for p*AtKASI:LjDIS* | SH115  SH117 | TTGGTCTCACGAAACCCTGATGATTATTAG  TGAGGTCTCGGAAATTAGGGCCTGAATGGAGC |
| *AtKASI* frag.1 cloning for p*DIS:AtKASI* | CG455  CG456 | ATGAAGACTTTACGGGTCTCACACCATGCAAGCTCTTCAATCTTCATCTCT  ATGAAGACTTGTCGCAAAGGTCGCGCATTG |
| *AtKASI* frag.2 cloning for p*DIS:AtKASI* | CG457  CG458 | ATGAAGACTTCGACGACAACAACGTTCCTTCA  ATGAAGACTTGAGTCCCATACCAGTAATGACAAC |
| *AtKASI* frag.3 cloning for p*DIS:AtKASI* | CG459  CG460 | ATGAAGACTTACTCGTCTCTGTGTTTGGTAACG  ATGAAGACTTTGGCTCTCTCCAAAACAAAATGTCA |
| *AtKASI* frag.4 cloning for p*DIS:AtKASI* | CG461  CG462 | ATGAAGACTTGCCACTAATTGTTGTATGCCCTAATAG  ATGAAGACTTCAGAGGTCTCACCTTTCAGGGTTTGAAGGCAGAGAAGGC |
| qPCR of *LjEF1alpha* | EF1alpha_F  EF1alpha_R | GCAGGTCTTTGTGTCAAGTCTT  CGATCCAGAACCCAGTTCT |
| qPCR of *LjKASI* | LjKASI_qPCR_F  LjKASI_qPCR_R | TCCCAACGCTAACTTCAAGC  CCCTGCATCATTGAGGCTAT |
| qPCR of *LjKASII* | AK42  AK43 | CGAGAAAGACTTGATCTCCCCAG  CGTGGTTACATCACTTGGTCATG |
| qPCR of *LjKASIII* | AK44  AK45 | GATTTGCATAGTAATGGTGATGG  GCATGAATATGAGGACTGCTTGG |
| qPCR of *LjDIS* | qPCR_DIS4_F  qPCR_DIS4_R | CATTCATTGATTTCGGGACA  CCAAACACAGAAGCAGATCAGA |
| qPCR of *LjDIS-like* | qPCR_DISL4_F  qPCR_DISL4_R | CATGTTATCGATTTGTGTTTGGA  TGACTACTACCCATTTGCTGAAAG |
| qPCR of *LjUbiquitin* | qPCR_F_LjUbi qPCR_R_LjUbi | ATGCAGATCTTCGTCAAGACCTT ACCTCCCCTCAGACGAAG |
| qPCR of *LjRAM2* | PP101  PP102 | ATCCTATGAGTGCACTAGCTTTACTAGAAG  AACGAGCAAATTAAAACTGAAAGAGAGTAC |
| qPCR for *LjSbtM1* |  | CACGTTGTTAGGACCCCAAT  TTGAGCAGCACCCTCTCTATC |
| qPCR for *LjBCP1* |  | TCATCTGTCCTTGGGGTCAT  CAGCTGCAGAAGTTGCATTT |
| qPCR for *LjPT4* |  | GAATAAAGGGGCCAAAATCG  GCTGTATCCTATCCCCATGC |
| qPCR for *LjAMT2.2* |  | TGGTTCAACTTTTCGTTCCA  CTTATCACCCTGACCCCAGA |
| qPCR for *LjSTR* |  | CTATATTGGTGACGAGGGAAGG  GTCCTGAGGTAGGTTCATCCAG |
| p*RAM2_1a* cloning for  p*RAM2:*g*RAM2* and  p*RAM2:GUS* | PP103  PP104 | ATGAAGACTTTACGGGTCTCAGCGGGATTGAAAGCTTCCCCATAG  TAGAAGACAAATCTTCTCCTAGTATTTTTTTTTTAAAG |
| p*RAM2_1b* cloning for  p*RAM2:*g*RAM2* and  p*RAM2:GUS* | PP105  PP106 | ATGAAGACTTAGATCATTCCACGGAGGAG  ATGAAGACTTCAGAGGTCTCACAGAGGTGAATGCACTTGTTGTTACTC |
| g*RAM2* cloning for  p*PT4:*g*RAM2/ram2-1* | AK20  AK21 | ATGAAGACTTTACGGGTCTCACACCATGGTGTCATCAACG  ATGAAGACTTCAGAGGTCTCACCTTGCAACCCATGACTTTGTTTG |
| p*RAM2* primer walking using TAC Lj T46c08 | PP132 | GTCGTTTTAGAAGAATTTTTTG |
| p*RAM2* primer walking using TAC Lj T46c08 | PP133 | AGGATAGGCTCAATACTTTGA |
| p*RAM2* primer walking using TAC Lj T46c08 | PP134 | ATGGGTGAAAGTGGTAAGATGG |
| p*RAM2* primer walking using TAC Lj T46c08 | PP135 | GCGTGACAAACATGGAAGG |
| p*RAM2* primer walking using TAC Lj T46c08 | PP136 | AGCAAAGTTGGGGGAGAAAT |
| p*RAM2* primer walking using TAC Lj T46c08 | PP137 | AGGTGGGTATTGGAGGTGGA |
| p*RAM2* primer walking using TAC Lj T46c08 | PP138 | ACACTTAAAAAAGAACGGAG |
| p*RAM2* primer walking using TAC Lj T46c08 | PP139 | CTCTAACAATCCACTATCTTG |
| p*RAM2* primer walking using TAC Lj T46c08 | PP140 | CACACAAGAACTTCATGCAC |
| p*RAM2* primer walking using TAC Lj T46c08 | PP141 | GAGCTTGATCACCTACTAATTAT |
| p*RAM2* primer walking using TAC Lj T46c08 | PP142 | CTTGTATGCCAGCAGCCTCAGAG |
| p*SbtM1 frag.1* cloning for pSbtM1:SPP-mCherry | JAVA-23  JAVA-24 | ATGAAGACTTTACGGGTCTCAGCGGAACATTGAGGACAGATTAAGG  TAGAAGACAATTGCCTTCATTTGTGCCAAA |
| p*SbtM1 frag.2* cloning for pSbtM1:SPP-mCherry | JAVA-25  JAVA-26 | TAGAAGACAAGCAAATAAACCGTCCAAGGC  ATGAAGACTTCAGAGGTCTCTCAGAGCTCCATCTTTAATTGGAATTTGATG |
| *SbtM1* secretion signal peptide cloning for pSbtM1:SPP-mCherry | SC278  SC279 | TATGGTCTCATCTGATGGAGCAAACCAAGTATAGGA  TATGGTCTCAGGTGTCATGCTCTTGGCCTTCCT |
