# Supplementary material for: Lipid transfer from plants to arbuscular mycorrhiza fungi
Source: eLife. 2017 Jul 20;6:e29107. doi: 10.7554/eLife.29107 (PMC5559270; doi:10.7554/eLife.29107)
Supplement: Supplementary file 3. — The Golden Gate toolbox is described in Binder et al. (2014). EV, empty vector; HR, hairy root; trafo, transformation. DOI: http://dx.doi.org/10.7554/eLife.29107.047 [file elife-29107-supp3.docx]

**Table S3:** **Plasmids used in this study**

Produced by classical cloning, Gateway cloning (Entry plasmids and Destination plasmids) and Golden Gate cloning (Level I, II and III). The Golden Gate toolbox is described in (48). EV, empty vector; HR, hairy root; trafo, transformation

| **Purpose** | **Name** | **Description** |
| --- | --- | --- |
| *dis-1* transgenic complementation  (Fig. 1A) | Entry: pENTR-p*DIS*:g*DIS* | PCR amplification of *DIS* promoter and gene with primers SH71 + SH72 and subcloning into pENTR/D-TOPO. |
|  | HR Trafo: p*DIS*:g*DIS* | LR clonase (Invitrogen) recombination of ENTR-p*DIS*:g*DIS* with pK7RWG2.0 w/o 35S promoter *(56)*. |
|  | HR Trafo: EV | Removal of Gateway casette from pK7RWG2.0 w/o 35S promoter (Antolín-Llovera et al., 2014) by EcoRV digest and religation. |
| Localization of *DIS* promoter activity (Fig. 2_S1) | p*DIS*-*GUS* | PCR amplification of 1.5 kb *DIS* promoter region with primers SH94 + SH104 and insertion into the HindIII and BamHI restriction sites of pBI101(Jefferson et al., 1987). |
| Cross species complementation *of Arabidopsis kasI* mutant with *LjDIS* (Fig. 4C) | Entry: pENTR-*pAtKASI:DIS:3’ UTR AtKASI* (pCG92)  Entry: pENTR-p*AtKASI*:*DIS*:3’ UTR *AtKASI* (pCG93)  *Arabidopsis* Trafo: p*KASI*:*DIS* (pCG94)  *Arabidopsis* Trafo: p*KASI*:*dis-1* (pCG95) | Assembled from L0 p*AtKASI*, L0 *DIS*, L0 *3’ UTR KASI* and pENTR-BsaI (BB04) by BsaI cut ligation  Assembled from L0 p*AtKASI*, L0 *dis-1*, L0 *3’ UTR KASI* and pENTR-BsaI (BB04) by BsaI cut ligation  LR clonase (Invitrogen) recombination of pCG92 with pMDC99 *(Curtis & Grossniklaus, 2003)*.  LR clonase (Invitrogen) recombination of pCG93 with pMDC99 (Curtis & Grossniklaus, 2003). |
| Localization of DIS in *N. benthamiana* leaves (Fig. 4F) | Entry: pENTR-*DIS* w/o stop  *N. benthamiana* Trafo: p*35S:DIS:RFP*  *N. benthamiana* Trafo: p*35S:AtLhcb1.3:YFP* | PCR amplification of *DIS* gene with primers SH93 + SH92 and subcloning into pENTR/D-TOPO.  LR clonase (Invitrogen) recombination of ENTR- pENTR-*DIS* w/o stop with pK7RWG2.0 (Karimi et al., 2002)*.*  LR clonase (Invitrogen) recombination of pENTR/D-TOPO-*AtLhcb1.3* w/o stop (kind gift from Jürgen Soll) with pB7FWG2.0 (Karimi et al., 2002). |
| Golden Gate level 0 and I (L0, LI) elements | | |
|  | L0 p*AtKASI* | PCR amplification of *AtKASI* 1.3 kb promoter fragment with SH113 + SH109 and assembly by StuI cut ligation into L0 pUC57 plasmid (BB01). |
|  | L0 *DIS* and  L0 *dis-1* | Assembled by StuI cut ligation into L0 pUC57 plasmid (BB01) from 2 PCR fragments amplified from genomic DNA of *L. japonicus* Gifu wild type (*DIS*) and *dis-1* mutant (*dis-1*). Primers:  Fragment 1: SH110 + SH 114  Fragment 2: SH115 + SH117 |
|  | L0 3’ UTR *AtKASI* | 3’ UTR of *AtKASI* (343 bp) was PCR amplified with primers SH118 + SH119 and assembled by StuI cut ligation into L0 pUC57 plasmid (BB01). |
|  | L0 p*RAM2A* | PCR amplification of 906 bp fragment *L. japonicus* Gifu genomic DNA with primers PP103+PP104. Assembly by SmaI cut ligation into LI-Amp (BB01) |
|  | L0 p*RAM2B* | PCR amplification of 1434 bp fragment *L. japonicus* Gifu genomic DNA with primers PP105+PP106. Assembly by SmaI cut ligation into LI-Amp (BB01) |
|  | LI A-C p*DIS* (pCG124) | PCR amplification of 1.5 kb *DIS* promoter from *L. japonicus* Gifu genomic DNA with primers SH122 + SH123 and BpiI cut ligation into LI-BpiI (BB03) plasmid. |
|  | LI C-D *AtKASI* (pCG125) | Assembled 4 PCR fragments amplified from *A. thaliana* Col-0 gDNA by BpiI cut ligation into LI-BpiI plasmid (BB03). Primers:  Fragment 1: CG455 + CG456  Fragment 2: CG457 + CG458  Fragment 3: CG459 + CG460  Fragment 4: CG461 + CG462 |
|  | LI A-B p*PT4* | PCR amplification of 2.2 kb *PT4* promoter region from *L. japonicus* Gifu genomic DNA with primers CG466 + CG467 and assembly by BpiI cut ligation into LI-BpiI plasmid (BB03). |
|  | LI C-D *DIS*  LI C-D *dis-1* | Assembled from two PCR amplified fragments from genomic DNA of *L. japonicus* Gifu wild type (*DIS*) and *dis-1* mutant (*dis-1*). Assembly by BpiI cut ligation into LI-BpiI plasmid (BB03). Primers:  Fragment 1: SH124 + SH138  Fragment 2: SH126 + SH127 |
|  | LI A-B *pRAM2* | Assembled by BpiI cut ligation from: L0 p*RAM2A* + L0 p*RAM2B* + LI-BpiI (BB03) |
|  | LI C-D *RAM2*  LI C-D *ram2-1* | PCR amplification of 1998 bp fragment *L. japonicus* Gifu genomic DNA with primers AK20 + AK21. Assembled by SmaI blunt end cut ligation: pUC57 (BB02) + Fragment: AK20 + AK21 |
|  | LI C-D *GUS* | (Pimprikar et al., 2016) |
|  | LI A-B p*SbtM1* | *PCR amplification of 559 bp fragment with primers JAVA-23 + JAVA-24 and of 211 bp fragment with primers JAVA-25 + JAVA-26 from pENTR D-TOPO pSbtM1. Assembled by BpiI cut ligation from 559 bp fragment + 211 bp fragment + LI-BpiI (BB03)* |
|  | LI B-C SSP (SbtM1 secretion signal peptide) | *PCR amplification of 135 bp fragment L. japonicus Gifu genomic DNA with primers SC278 + SC279. Assembly by SmaI cut ligation into LI-pUC57 (BB02)* |
| Golden Gate level II (LII) plasmids | | |
|  | LII R 3-4 p*35S:mCherry* (selection marker for HR) | Assembled by BsaI cut ligation from:  LI A-C p*35S* (G009) + LI C-D mCherry (G057) + LI dy D-E (B008) + LI E-F 35S-T (G059) + LI dy F-G (BB09) + LII R 3-4 |
|  | LII F 1-2 p*DIS*:*AtKASI* (pCG126) | Assembled by BsaI cut ligation from:  LI A-C p*DIS* + LI C-D *AtKASI* + LI dy D-E (BB08) + LI E-F nos-T(G006) + LI dy F-G (BB09) + LIIc F 1-2 (BB30) |
|  | LII F 1-2 p*DIS*:*EV* (pCG127) | Assembled by BsaI cut ligation from:  LI A-C p*DIS* + LI dy C-D (BB07) + LI dy D-E (BB08) + LI E-F nos-T(G006) + LI dy F-G (BB09) + LIIc F 1-2 (BB30) |
|  | LII F 1-2 p*PT4*:*DIS* (pCG130) | Assembled by BsaI cut ligation from:  LI A-B p*PT4* + LI dy B-C (BB06) + LI C-D *DIS* + LI E-F nos-T(G006) + LI dy F-G (BB09) + LIIc F 1-2 (BB30) |
|  | LII F 1-2 p*PT4*:*dis-1* (pCG131) | Assembled by BsaI cut ligation from:  LI A-B p*PT4* + LI dy B-C (BB06) + LI C-D *dis-1*+ LI E-F nos-T(G006) + LI dy F-G (BB09) + LIIc F 1-2 (BB30) |
|  | LIIc F 1-2 p*RAM2:gRAM2*  pPP106 | Assembled by BsaI cut ligation from: LI A-B p*RAM2* + LI B-C dy (BB06) + LI C-D *RAM2* + LI D-E dy (BB08) + LI E-F nos-T (G006) + LI F-G dy (BB09) + LIIc F 1-2 (BB30) |
|  | LII F 3-4 *pPT4:gRAM2*  (pAK12) | Assembled by BsaI cut ligation from:  LI A-B p*PT4* + LI dy B-C (BB06) + LI C-D *RAM2* + LI E-F nos-T(G006) + LI dy F-G (BB09) + LIIc F 3-4 (BB34) |
|  | LII F 3-4 *pPT4:gram2-1*  (pAK13) | Assembled by BsaI cut ligation from:  LI A-B p*PT4* + LI dy B-C (BB06) + LI C-D *ram2* + LI E-F nos-T(G006) + LI dy F-G (BB09) + LIIc F 3-4 (BB34) |
|  | LIIc F 1-2 p*RAM2:GUS*  pPP107 | Assembled by BsaI cut ligation from: LI A-B p*RAM2* + LI B-C dy (BB06) + LI C-D GUS + LI D-E dy (BB08) + LI E-F nos-T (G006) + LI F-G dy (BB09) + LIIc F 1-2 (BB30) |
|  | LIIc R 3-4 p*Ubi:mCherry*  (pPP101) | *(Pimprikar et al., 2016)* |
|  | LIIβ F 5-6 p*POI:NLS-2XYFP:NosT*  (pGC134) | Assembled by BsaI cut ligation from: LI A-B Esp3I-*lacZ* dy (G082) + LI B-C NLS (G60) + LI C-D YFP (G54) + LI D-E YFP (G12) + LI E-F Nos-T (G006) + LI F-G dy (BB09) + LIIβ F 5-6 (BB28) |
|  | LIIc F 1-2 p*SbtM1:SPP-mCherry: HspT*  (pPP137) | Assembled by BsaI cut ligation from: LI A-B p*SbtM1* + LI B-C SPP + LI C-D *mCherry* + LI D-E dy (BB08) + LI E-F Hsp-T (G045) + LI F-G dy (BB09) + LIIc F 1-2 (BB30) |
| Golden Gate level III (LIII) plasmids for plant transformation | | |
| *ram2-1* transgenic complementation (Fig. 1A) | LIIIβ F A-B p*RAM2:RAM2*  (pPP162) | Assembled by BpiI cut ligation from: LIIc F 1-2 p*RAM2:RAM2* + LII 2-3 ins (BB43) + LIIc R 3-4 p*Ubi:mCherry* + LII 4-6 dy (BB41) + LIIIβ F A-B (BB53) |
| Localization of *DIS* promoter activity (Fig 2_S1) | LIIIβ F A-B p*DIS:GUS*  (pMP2) | Assembeled by Esp3I Cut-Ligation. PCR product of p*DIS* + pPP170 [LIIc F 1-2 p*RAM1:GUS* + LII 2-3 ins (BB43) + LIIc R 3-4 p*Ubi:mCherry* + LII 4-6 dy (BB41) + LIIIβ F A-B (BB53)] (Pimprikar et al., 2016). |
| Localization of *RAM2* promoter activity (Fig 2_S1) | LIIIβ F A-B pRAM2*:GUS*  (pPP163) | Assembled by BpiI cut ligation from: LIIc F 1-2 p*RAM2:GUS* + LII 2-3 ins (BB43) + LIIc R 3-4 p*Ubi:mCherry* + LII 4-6 dy (BB41) + LIIIβ F A-B (BB53) |
| Cross species complementation *of dis-1* mutant with *Arabidopsis KASI* (Fig. 2C) | LIIIβ F A-B p*DIS*:*AtKASI* (pCG128)  LIIIβ F A-B p*DIS*:*EV* (pCG129) | Assembled by BpiI cut ligation from:  LII F 1-2 p*DIS*:*AtKASI* + LII ins 2-3 (BB43) + LII R 3-4 p35S:mCherry + L II dy 4-6 (BB41) + LIIIβ F A-B  Assembled by BpiI cut ligation from:  LII F 1-2 p*DIS*:*EV* + LII ins 2-3 (BB43) + LII R 3-4 p35S:mCherry + L II dy 4-6 (BB41) + LIIIβ F A-B |
| *dis-1* transgenic complementation with p*PT4*:*DIS*  (Fig. 2C) | LIIIβ F A-B p*PT4*:*DIS* (pCG132)  LIIIβ F A-B p*PT4*:*dis-1* (pCG133) | Assembled by BpiI cut ligation from:  LII F 1-2 p*PT4*:*DIS* + LII ins 2-3 (BB43) + LII R 3-4 p35S:mCherry + L II dy 4-6 (BB41) + LIIIβ F A-B  Assembled by BpiI cut ligation from:  LII F 1-2 p*PT4*:*dis-1* + LII ins 2-3 (BB43) + LII R 3-4 p35S:mCherry + L II dy 4-6 (BB41) + LIIIβ F A-B |
| *ram2-1* transgenic complementation with p*PT4*:RAM2  (Fig. 2C) | LIIIβ F A-B p*PT4*:*RAM2* (pAK14)  LIIIβ F A-B p*PT4*:*ram2-1* (pAK15) | Assembled by BpiI cut ligation from:  LII F 1-2 p*Ubi:mCherry* + LII ins 2-3 (BB43) + LII F 3-4 p*PT4:gRAM2* + L II dy 4-6 (BB41) + LIIIβ F A-B  Assembled by BpiI cut ligation from:  LII F 1-2 *pUbi:mCherry* + LII ins 2-3 (BB43) + LII F 3-4 p*PT4:ram2-1* + L II dy 4-6 (BB41) + LIIIβ F A-B |
| Esp3I compatible destination backbone for Localization of promoter activity | Esp3I cut ligation compatible backbone: LIIIβ F A-B p*SbtM1:SP-mCherry_pPOI:NLS-2XYFP* (pPP217) | Assembled by BpiI cut ligation from: LIIc F 1-2 *pSbtM1*:*SP*-*mCherry*: HspT + LII 2-3 ins (BB43) + LII 3-4 dy (BB64) + LII 4-5 ins (BB44) + LIIβ F 5-6 *pPOI:NLS-2XYFP:NosT* + LIIIβ F A-B (BB53) |
| BsaI compatible destination backbone for Localization of promoter activity | BsaI cut ligation compatible backbone: LIIIβ F A-B p*SbtM1:SP-mCherry_*p*POI:NLS-2XYFP* (pPP218) | Assembled by Esp3I cut ligation from: LIIIβ F A-B *pSbtM1:SP-mCherry*_*pPOI:NLS-2XYFP* + LI A-B Esp3I-ccdB dy (G084) |
| Localization of promoter activity of *pDIS* (Fig 2A) | LIIIβ F A-B p*SbtM1:SSP:mCherry+pDIS:NLS-2xYFP* (pPP241) | Assembled by BsaI cut ligation from:  LI A-B pDIS + LIIIβ F A-B p*SbtM1:SP-mCherry_*p*POI:NLS-2XYFP* (pPP218) |
| Localization of promoter activity of *pRAM2* (Fig 2B) | LIIIβ F A-B p*SbtM1:SSP:mCherry+pRAM2:NLS-2xYFP* (pPP238) | Assembled by BsaI cut ligation from:  LI A-B pRAM2 + LIIIβ F A-B p*SbtM1:SP-mCherry_*p*POI:NLS-2XYFP* (pPP218) |

**References**

Antolín-Llovera M, Ried Martina K, Parniske M. 2014. Cleavage of the SYMBIOSIS RECEPTOR-LIKE KINASE ectodomain promotes complex formation with Nod Factor Receptor 5. *Current Biology* **24**(4): 422-427. 10.1016/j.cub.2013.12.053.

Curtis MD, Grossniklaus U. 2003. A gateway cloning vector set for high-throughput functional analysis of genes *in planta*. *Plant Physiology* **133**(2): 462-469. 10.1104/pp.103.027979.

Jefferson RA, Kavanagh TA, Bevan MW. 1987. GUS fusions: beta-glucuronidase as a sensitive and versatile gene fusion marker in higher plants. *EMBO J* **6**: 3901-3907.

Karimi M, Inzé D, Depicker A. 2002. GATEWAY™ vectors for *Agrobacterium*-mediated plant transformation. *Trends in Plant Science* **7**(5): 193-195. 10.1016/S1360-1385(02)02251-3.

Pimprikar P, Carbonnel S, Paries M, Katzer K, Klingl V, Bohmer M, Karl L, Floss D, Harrison M, Parniske M, et al. 2016. A CCaMK-CYCLOPS-DELLA complex regulates transcription of RAM1, a central regulator of arbuscule branching. *Current Biology* **26**: 987-998. 10.1016/j.cub.2016.01.069.
